# Endoscopic, Ultrasonographic, and Histologic Descriptions of Dextranomer/Hyaluronic Acid in a Case of Fecal Incontinence

**DOI:** 10.1155/2018/5873094

**Published:** 2018-07-26

**Authors:** Trent Irwin, Alexandria R. Snow, Taylor S. Orton, Christie Elliott

**Affiliations:** University of Nevada, Reno School of Medicine, 1664 North Virginia Street, Reno, NV 89557-0357, USA

## Abstract

**Objective:**

To present a case of fecal incontinence treated with dextranomer/hyaluronic acid (Solesta®) injections, which later caused clinical confusion and avoidable interventions. The endoscopic, ultrasonographic, and histologic appearances of dextranomer/hyaluronic acid will also be reported.

**Case Presentation:**

A middle-aged Hispanic male who failed conservative management of his fecal incontinence was injected with dextranomer/hyaluronic acid in an attempt to alleviate symptoms. An unrelated screening colonoscopy was performed soon after, revealing a submucosal rectal lesion. Flexible sigmoidoscopy and endoscopic rectal ultrasound with FNA were scheduled for patient for further evaluation. An unknown foreign material was noted under microscopy and, upon attaining additional history, the gastroenterologist uncovered the patient's recent injections of dextranomer/hyaluronic acid.

**Conclusion:**

Dextranomer/hyaluronic acid for the treatment of fecal incontinence has become more common in recent years. Though the imaging and histologic appearance of this gel-like material is seen in other areas of medicine, equivalent descriptions are limited in the anorectal region. To curb misdiagnoses and prevent unnecessary interventions, it is important to expound on the endoscopic, imaging, and histopathologic features of this tissue-bulking agent in the setting of fecal incontinence and to encourage communication, proper documentation, and easy accessibility to patient health information by all medical staff.

## 1. Introduction

Dextranomer/hyaluronic acid (Solesta) is an injectable, biocompatible bulking agent which may be used for the treatment of fecal incontinence. Though dextranomer/hyaluronic acid (DxHA) has been used in the pediatric population for treating vesicoureteral reflux (Deflux®), it was not until 2011 that the FDA approved its use in treating adult fecal incontinence in those who have failed conservative therapy [1]. While there are multiple case reports describing DxHA misdiagnoses, imaging appearances, and histopathology in the genitourinary system, there are a limited number of cases reports which reference DxHA in the anorectal region. In fact, in a literature review that was undertaken via PubMed with search terms “Solesta”, “dextranomer/hyaluronic acid”, and “dextranomer/hyaluronic acid and fecal incontinence”, a paper describing the histopathology of DxHA when used for fecal incontinence could not be found.

Defined as the involuntary loss of solid or liquid feces, fecal incontinence (FI) affects anywhere from 2% to 18% of the general population [2]. This figure is likely higher, as the shame and emotional toll of this condition assumedly lead to underreporting. The initial treatments to combat FI consist of supportive care and medical therapies. If conservative measures fail and anorectal manometry or endorectal imaging show no abnormalities, then DxHA is often the next line of treatment in FI [1].

As DxHA becomes more commonly used, it is necessary for physicians to ascertain if a patient with a history of FI has been treated with an injectable bulking agent. An understanding of the endoscopic appearance, imaging features, and microscopy of DxHA is also important in limiting unnecessary interventions and clinical confusion. This report will discuss the case of a middle-aged male who had a fine-needle aspiration (FNA) biopsy following sigmoidoscopy and endoscopic ultrasonography, which showed features consistent with a history of DxHA treatment.

## 2. Case Presentation

A 41-year-old Hispanic male (BMI: 44.6) presented to the emergency department after seven days of severe, novel, left-sided rectal pain. The patient denied fever, nausea, vomiting, bowel changes, or signs of blood in his stool. Though a proper rectal exam could not be performed due to pain, a 6 mm mass with surrounding erythema was noticed adjacent to the rectum in the 4 o'clock position. A diagnosis of perirectal abscess was made and incision and drainage were performed. One week after the procedure, the patient described a “ripping” sensation during a large bowel movement that led to worsening of his rectal pain. He was referred to a colorectal surgeon for presumed anal fissure, but confirmatory rectal exam was not possible due to physical discomfort. A subsequently scheduled rectal exam under general anesthesia was cancelled by the patient, and he was lost to follow-up.

During an unrelated consult for weight-loss surgery three months later, the bariatric surgeon discovered that the patient had new onset pruritus ani for nearly one month. Evidence of anorectal pain, hemorrhoids, fissures, or fistulas were absent at this time. Patient was prescribed lidocaine 5% topical ointment for two weeks PRN. Four months later, at the patient's request, the bariatric surgeon rechecked for the possibility of an anal fissure. Between these office visits, patient continued to have rectal pain (though of diminishing severity), bright red blood on toilet paper, pruritus ani, blood in his semen, loose stools, and the onset of outright FI. Patient attempted self-treatment of his FI with stool-bulking agents, fiber, psyllium, lidocaine cream, and Sween Cream, but with no relief. As these conservative measures failed, it was determined that anorectal manometry was warranted, but this test showed no abnormalities. Both the physician and patient decided that DxHA injections were the next best option. One week later, 4 X 1 mL injections of DxHA were administered approximately 5 mm above the dentate line at the posterior, anterior, left lateral, and right lateral positions without complications.

**Figure 1:**
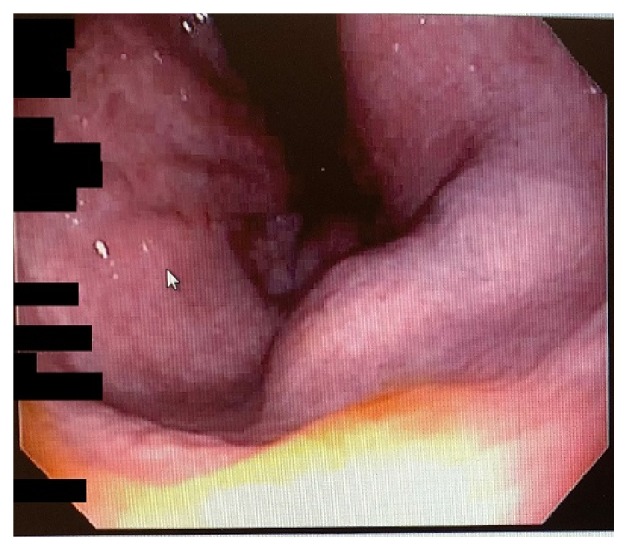
Endoscopic view approximately 6-7 cm from the anal verge showing a 15 mm bulging subepithelial lesion.

One month following DxHA injections, the patient underwent a previously scheduled screening colonoscopy because of a significant family history of colon cancer in his mother (diagnosed at 58 years; died at 62 years). A 5 mm sessile serrated polyp in the ascending colon and a 3 mm hyperplastic polyp in the sigmoid colon were removed during this procedure, but it was also noted by the gastroenterologist that a 15 mm benign-appearing submucosal lesion was present in the distal rectum (~5 cm from the anus). Unsure of what this lesion could be, a flexible sigmoidoscopy and endoscopic ultrasound were ordered to be performed two weeks later. On flexible sigmoidoscopy, the bulging submucosal lesion was again noted ~6-7 cm from the anal verge (Figure 1) and endoscopic ultrasound showed this mass to be a homogenous, hypoechoic lesion (0.79 cm X 2.98 cm) that was contiguous with the muscularis propria. Fine-needle aspiration of the rectal lesion was performed and sent to pathology.

**Figure 2:**
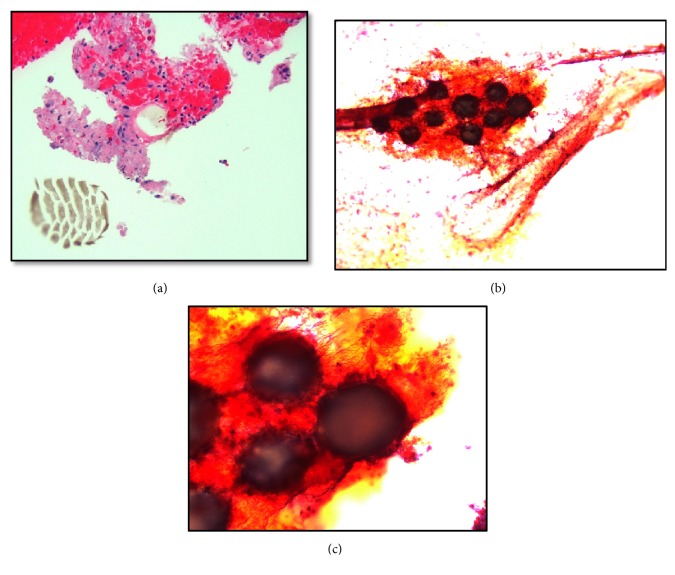
**(a)** Rectal FNA biopsy (H&E, 20X) with strips of benign colonic mucosa and the presence of giant cells, reactive macrophages, acute inflammatory cells, mucin, and foreign material.** (b)** Dark blue/purple dextranomer microspheres and hyaluronic acid (10X).** (c)** Dark blue/purple dextranomer microspheres and hyaluronic acid (40X).

Under microscopy, the hospital pathologist described the rectal FNA samples as having clusters of reactive macrophages, acute inflammation, giant cells, mucin, and multiple, spherically shaped dark microparticles (Figures 2(a)–2(c)). Though noted as clearly foreign by the pathologist, the presence and etiology of these particles were perplexing. After calling the gastroenterologist to describe these findings, additional history attained by the gastroenterologist from the patient revealed the recent history of DxHA injections. Thus, it was surmised that the rectal lesion and corresponding histopathology were both a result of the patient's FI treatment.

Unfortunately, though there was initial improvement with the DxHA injections, at the two-month follow-up appointment the patient described worsened FI (several episodes daily, especially after bowel movements). As of this report, supportive measures, biofeedback training, and topical ointments were being used to treat the patient's incontinence.

## 3. Discussion

Conservative measures are the first line of treatment in patients with FI. This includes dietary or behavioral modifications, biofeedback, pads, perianal skin care, fiber supplementation (loose stool FI), antidiarrheal medications (liquid stool FI), and enemas (overflow FI) [1]. Though other surgical treatments are available if conservative measures fail (e.g., sphincteroplasty, dynamic graciloplasty, and sacral nerve stimulators), treatment for FI with DxHA injections has become increasingly popular. In this outpatient procedure, four equally spaced 1 mL injections are administered 5 mm proximal to the dentate line, without the need for anesthesia or sedation [3]. Though no DxHA studies show 100% improvement in FI, four prospective studies (n=419) have shown a response rate of 56-61% of patients within 12-20 months of follow-up (successful response was defined as ≥50% reduction in weekly FI episodes). Greatest success was seen in those with mild to moderate FI, but many patients did require multiple injections to achieve clinical improvement [4].

Though relatively new to FI treatment, DxHA has been studied and clinically used in other areas of medicine. DxHA injections into the pharynx, vocal cords, skin, and gastroesophageal junction (in animal studies) are all present in the literature [5–9]. Since FDA approval in 2001, DxHA (Deflux) has been commonly used in the pediatric population to treat vesicoureteral reflux [10]. By injecting this bulking agent below the ureteral orifice of the bladder, urine reflux is stopped and ureteral reimplantation surgery can be avoided [11]. There have been many vesicoureteral reflux (VUR) cases in the literature which created clinical confusion, unnecessary interventions, and even misdiagnoses in patients with previous histories of DxHA injections. Multiple reports have described calcified DxHA mimicking distal ureteral calculi, sometimes leading to cystoscopy or ureteroscopy [12–15]. In one case, a patient with a past history of VUR was referred by her gynecologist to the pediatric urologist after misdiagnosing DxHA implants on transvaginal ultrasonography as a bladder tumor [10]. After further history and repeat ultrasound, the ellipsoid hyperechoic submucosal masses were concluded to be merely remnants of her DxHA injections. Similar to the patient in this report, an incomplete history and diagnostic test mimicry led to an unnecessary workup in this case.

The microscopic features of DxHA have been described previously in the VUR literature, but it should be noted that a histopathologic case of DxHA at the anorectal region could not be found by the authors. Consisting of dextranomer microspheres (80-250 *μ*m) within a viscous matrix of stabilized sodium hyaluronate, DxHA is known to promote collagen and fibroblast formation between individual microspheres, leading to a submucosal bulking effect [3]. Related to this function, in clinical cases of VUR, the most common findings have been purple-blue dextranomer microspheres, a blue-gray amorphous material (hyaluronic acid), granulomatous reactions with multinucleated giant cells, inflammatory infiltrates (i.e., lymphocytes, plasma cells), pseudoencapsulation of microspheres with fibrosis, and sometimes calcification [11, 16, 17]. In an intriguing animal study that followed the histological changes of rat bladders (n=30) at the three-, six-, and twelve-month periods, similar pathologic findings were also noted. By twelve months into this study, inflammatory infiltration and fibroblast formation were most prevalent (100%), followed by foreign body type giant cells (76%), capillary growth (67%), and granuloma formation (43%). Interestingly, granuloma formation was not seen in any of the sacrificed three-month rats but was seen in 50% of the six-month rats and 80% of the twelve-month rats [18]. Similar microscopic changes have also been noted in the skin of a patient who was treated for periorbital wrinkles [9]. The patient's rectal FNA in this report corresponded to the preceding cases in that multiple dextranomer microspheres within a mucinous material were present. Acute inflammation, reactive macrophages, and giant cells were also seen.

Similar to the histopathology of DxHA, endoscopic and imaging descriptions are limited in the anorectal region. One case concerning the endoscopic and computed tomography (CT) appearance of DxHA (Solesta) injections was found in the literature [19]. An elderly woman who had undergone endoscopic DxHA injections for FI subsequently had an abdominopelvic CT scan two days later. The endoscopic view showed the submucosal bulking property of DxHA clearly. The CT was read as having mural rectal thickening with multiple round hypodense foci within the rectal wall. Prior to the DxHA injection history being known, mucinous mural adenocarcinoma and abscess were among the radiological differential diagnoses. This case further illustrates the importance of a thorough history to prevent misdiagnoses and unnecessary interventions. Likewise, the unusual rectal findings seen at screening colonoscopy in this report's patient steered clinicians towards interventions that could have been avoided had the history of DxHA injections been known.

## 4. Conclusion

As anorectal DxHA injections continue to be used in the treatment of FI, it is particularly important for physicians to be familiar with how this bulking agent appears in various settings. Though DxHA has been elucidated in the VUR literature, further research concerning the endoscopic, radiologic, and histopathologic characteristics of DxHA in the context of FI is necessary. Effective communication between patients who have a history of FI with DxHA treatment and other clinicians is also important to reduce potential medical errors. Likewise, proper documentation and ease of access to health information by all medical staff involved in the patient's care will help to avoid future clinical uncertainty, misdiagnoses, and needless interventions. This case report is meant to bring further awareness in a step towards that goal.
